# Response Mechanisms of Bacterial Degraders to Environmental Contaminants on the Level of Cell Walls and Cytoplasmic Membrane

**DOI:** 10.1155/2014/873081

**Published:** 2014-06-26

**Authors:** Slavomíra Murínová, Katarína Dercová

**Affiliations:** ^1^Department of Biochemical Technology, Faculty of Chemical and Food Technology, Institute of Biotechnology and Food Science, Slovak University of Technology, Radlinského 9, 812 37 Bratislava, Slovakia; ^2^Water Research Institute, Nábrežie arm. gen. L. Svobodu 5, 812 49 Bratislava, Slovakia

## Abstract

Bacterial strains living in the environment must cope with the toxic compounds originating from humans production. Surface bacterial structures, cell wall and cytoplasmic membrane, surround each bacterial cell and create selective barriers between the cell interior and the outside world. They are a first site of contact between the cell and toxic compounds. Organic pollutants are able to penetrate into cytoplasmic membrane and affect membrane physiological functions. Bacteria had to evolve adaptation mechanisms to counteract the damage originated from toxic contaminants and to prevent their accumulation in cell. This review deals with various adaptation mechanisms of bacterial cell concerning primarily the changes in cytoplasmic membrane and cell wall. Cell adaptation maintains the membrane fluidity status and ratio between bilayer/nonbilayer phospholipids as well as the efflux of toxic compounds, protein repair mechanisms, and degradation of contaminants. Low energy consumption of cell adaptation is required to provide other physiological functions. Bacteria able to survive in toxic environment could help us to clean contaminated areas when they are used in bioremediation technologies.

## 1. Introduction

Over hundreds of years, mankind has been producing millions of tons of dangerous pollutants. A significant part of this pollution consists of hydrophobic organic compounds that are extremely persistent. These compounds have been stored in soil and water sediments and tend to persist unmodified over decades. Nowadays, there is a growing awareness concerning the toxic or even carcinogenic effects of these chemicals. Efficient ways to dispose this waste are physical and chemical techniques that include combustion, photolysis, chemical degradation, and decomposition. Each chemical method can be successfully applied only within a certain range of concentrations of organic compounds due to their solubility, toxicity, and persistence [1]. Limitations of the application of these techniques in the environment are caused by low concentration of the pollutants. Alternative methods for the decontamination are represented by biodegradation or phytoremediation. Biodegradation has long been seen as a cost-effective and ecological way to eliminate environmental contamination [2]. However, the toxicity of the chemicals can hamper application of microorganisms for removal of the pollutants. Bacteria used for biodegradation must be able to survive and colonize the contaminated area. Some bacteria have developed efficient adaptation mechanisms to survive under adverse conditions [3–5]. Anomalies in environmental conditions activate in cells a series of processes that allow microorganisms to minimize their negative impact. All adaptation mechanisms are synchronized to ensure necessary physiological functions with low energy consumption. Environmentally induced perturbations in cell membrane structure may result in significant disturbance of some physiological functions. Flexibility and adaptation capacity of the membrane largely determines survival of the cells [6, 7]. Since membranes constitute the main target for the action of solvents, most adaptive mechanisms are concerned with maintenance of the membrane fluidity and lipid-phase stability [8]. Fluidity of cytoplasmic membrane is a very important characteristic of the membrane structure and is defined as the reciprocal value of its viscosity. It can be modulated by the alteration of fatty acids that build membrane phospholipids.

## 2. Cytoplasmic Membrane

Cytoplasmic membrane is a dynamic structure, which consists of stable phospholipid bilayer with motile proteins. The lipid components of the membrane form a barrier to the transport of molecules, while protein components act as transport structures of pumps and channels that allow selected molecules to circulate into and out of the cell [9]. Cytoplasmic membranes of most bacterial strains consist of phosphatidylethanolamine—PE (75%), phosphatidylglycerol—PG (15–20%), and cardiolipin—CL (5–10%). Unlike mammals, only a few bacterial species contain phosphatidylcholine (PC) in the membranes (e.g.,* Rhodopseudomonas sphaeroides, Pseudomonas stutzeri*). These bacteria tend to be highly specialized or highly evolved. PC is synthesized by three successive methylations of PE [10].

Studies with* E. coli *showedthat bacterial phospholipids are synthesized exclusively for use in the biogenesis of membranes [11]. The enzymes of fatty acid biosynthesis are located in the cytoplasmic membrane, and the enzymes that metabolize phospholipids are bound to the inner part of the membrane. Biosynthesis of one mole of phospholipid from acetyl-CoA and sn-glycerol-3-phosphate requires 32 mol of ATP. Phospholipids constitute 10% of the dry weight of the cell, which means that a significant amount of energy expended in the biogenesis of a new cell is used in the production of membrane phospholipids [10, 12].

The diversity of cell membrane functions is conditioned by their structure. Despite their different functions, the membranes have according to Berg et al. [13] in common several similarities:

1. membranes are sheet-like structures with the thickness between 6 nm and 10 nm, 60–100 Å, respectively. They form dosed boundariesbetween different compartments [13];
2. membranes consist mainly of lipids and proteins with or without carbohydrate appendix. The mass ratio of lipids to proteins ranges from 1 : 4 to 4 : 1 [14];
3. membrane phospholipids are molecules with average size about 10 angström. They have hydrophilic and hydrophobic moieties. Because of the differences in polarity of their constituents, they spontaneously form bimolecular sheets in aqueous environment—membrane bilayers. The bilayers of lipids are barriers to the flow of polar molecules [15];
4. membranes contain specific proteins, which mediate distinctive functions of particular membranes. Membrane proteins can have various functions, for example, receptors, membrane pumps, channels, energy transducers, and enzymes. Membrane proteins can be fully embedded in lipid bilayers or may stick out from the membrane structure [16];
5. membranes are noncovalent cell components. The most abundant membrane elements, proteins, and phospholipids are linked together by noncovalent interactions, which act cooperatively [16, 17];
6. membranes are in general asymmetric. The two faces of biological membranes always differ from each other [14];
7. the fluidity is the most important membrane characteristic. Lipid molecules diffuse rapidly in the plane of the membrane, as do proteins, unless they are bonded by specific interactions. In contrast, lipid molecules and proteins do not readily rotate across the membrane. Membranes can be regarded as two-dimensional bilayers of oriented proteins and lipids [13];
8. most cell membranes are electrically polarized with a negatively charged inside (typically 60 millivolts). Membrane potential plays a key role in transport, energy conversion, and excitability [13, 14, 16, 17].

The most important function of cytoplasmic membrane of bacteria is to form a permeable barrier, regulating the passage of solutes between the cell and the outer environment. The membrane keeps essential metabolites and macromolecules inside the cell, it pumps nutrients into the cell against a concentration gradient, and it prevents the entry of certain compounds present in the environment [12, 18]. The barrier properties of the cytoplasmic membrane are of special importance for the energy transduction of the cell [19].

### 2.1. Mechanism of Disturbance of the Cytoplasmic Membrane by Organic Compounds

Many organic pollutants are able to penetrate into cytoplasmic membrane resulting in swelling of the membrane and increase of membrane fluidity. This increase leads to the loss of membrane functionality and to the damage of bacterial cell. Heipieper et al. [3] established the existence of a systematic relationship between the values of log P in the range 1–5 and values for the partitioning of solvents in cytoplasmic membrane. When a solvent penetrates into a membrane, it disturbs the integrity of that membrane and, hence, its function leads to an uncontrolled proton and potassium ions efflux. This leakage causes a lowering in the proton-motive force and leads to an impairment in the energy conservation [20, 21]. The experiments with nine solvents (benzene, toluene, ethylbenzene, o-xylene, cyclohexane, naphthalene, biphenyl, *α*-pinene, and decalin), each with log *P* values between 2 and 5, confirmed that their concentration in the membrane of up to 0.5 *μ*mol*·*mg^−1^ phospholipid resulted in an increase in the surface area of the membrane [21]. This partitioning level corresponds with approximately one solvent molecule per two phospholipid molecules. As a consequence, membrane fluidity is affected. Bacterial cells try to undertake appropriate responses to minimize disruptive effect of organic compounds by readjustment of fluidity.

## 3. Adaptation Mechanisms of Bacterial Strains

Membrane fluidity is the most important parameter that determines cell viability. Bacterial cells can change the fluidity of this surface component depending on the outer conditions. This could be performed by the alteration of fatty acid composition, their chain length, and phospholipid composition [22]. Most bacteria are resisting the fluidizing effect of hydrophobic compounds by changing their membrane composition to reduce fluidity and to maintain balance between bilayer and nonbilayer forming phospholipids [23]. These adaptation mechanisms rely on a modification of the membrane phospholipids. The alterations in the cytoplasmic membrane composition play crucial role in adaptation to the presence of high concentrations of toxic contaminants. Changes in the fatty acid composition of membrane lipids are the most important reactions of bacteria against membrane active substances. Most adaptive mechanisms were only described for Gram-negative (G^−^) aerobic bacteria [19, 24–28]; however, some papers describing Gram-positive (G^+^) aerobic [29–36] and anaerobic bacteria are also available [37, 38]. The last mentioned group of bacteria is rarely studied. The first systematic approach of anaerobic bacteria was published by Duldhardt et al. [38]. It demonstrates that anaerobic bacteria were about three times more sensitive towards a series of different organic compounds when compared with aerobic bacteria. This behavior was explained by a lower growth rate of anaerobic bacteria in comparison with aerobic species.

### 3.1. Changes in Fatty Acids Composition

#### 3.1.1. Rigidification of Cytoplasmic Membrane

**Figure 1:**
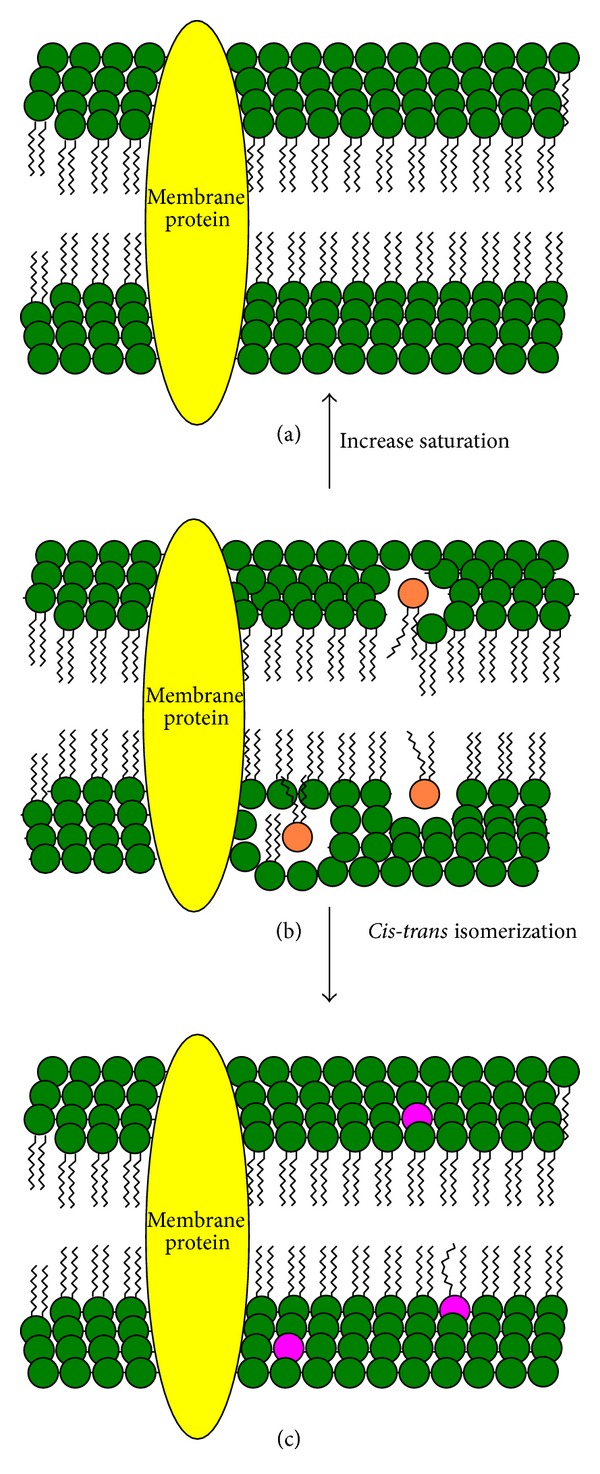
Two mechanisms increasing bacterial membrane saturation and decreasing membrane fluidity. The first one (direction from (b) to (a)) describes the increase of the synthesis of saturated fatty acids (green circles) instead of* cis* unsaturated fatty acids (orange circles); the second one (from (b) to (c)) shows the isomerization of* cis* unsaturated fatty acids into corresponding* trans* isomers (purple circles).

Segura et al. [39] mentioned that the alteration of the ratio of long-chain to short-chain fatty acids is involved in the regulation of the membrane fluidity under adverse conditions. Larger share of long-chain fatty acids can impede the pollutant penetration into the membrane. This results in lower concentration of the pollutant in the membrane and, thus, reduces its toxicity. However, increase of membrane saturation (rigidification) is a more efficient mechanism. Increase in saturation of membrane phospholipids in the presence of toxic organic compounds has been described in several publications [25, 26, 40, 41]. The IC_50_–IC_75_ concentration of toxic compound leads to the highest increase in membrane saturation [3, 38]. Previous reports indicated that aromatic compounds such as benzene, biphenyl, phenol, PCBs, and toluene can accumulate in membrane bilayer between the acyl chains of fatty acids. This phenomenon leads to the higher membrane fluidity. Bacterial cells try to counteract this effect with closer packing of fatty acid alkyl chains of the phospholipids in the cell membrane to increase the membrane rigidity and prevent the solvent accumulation [19, 21, 38, 42]. The same mechanism was observed in* Pseudomonas stutzeri* in the presence of naphthalene. The increasing degree of membrane lipid saturation is one of the major adaptive mechanisms of bacteria cells to the presence of many aromatic compounds [3, 43, 44]. This alteration helps cells survive under long-term adverse conditions. The reasons for the ability of tightly packing saturated fatty acids are their spherical conformation (Figure 1(a)) and high phase transition temperatures (*T*_*M*_). Weber and de Bont [45] characterized the transition from the ordered phase (gel) into disordered phase (liquid-crystalline) (*T*_*M*_) and described the location of phospholipids in membrane. *T*_*M*_ for long-chain saturated fatty acids are very high (e.g., for palmitic acid, it is 63°C). This means that palmitic acid stays in ordered phase below 63°C. Similarly, other long-chain saturated fatty acids contribute to low fluidity of membrane and increase membrane ordering under growth temperatures. This arrangement prevents fluidizing compounds to accumulate in membrane fractions. The corresponding monounsaturated fatty acids have lower *T*_*M*_. The lowest *T*_*M*_ was measured for unsaturated fatty acids with* cis *configuration of double bond; for example, for C16:1*cis*, it was 0°C; for C16:1*trans*, it was 33°C [46–48]. Bacterial cells try to increase membrane rigidity to counteract the fluidization effect of organic pollutants. This will be provided with specific amount of fatty acids contributing to a lower *T*_*M*_ and others to a higher *T*_*M*_. The average composition will result in an average *T*_*M*_.

The mechanism of increase of saturation degree has limitation due to the condition of synthesis of saturated fatty acids. In G^−^ bacteria, only the energy-dependent* de novo *biosynthesis of saturated fatty acids allows for an increase in the degree of saturation with increasing the proportion of saturated to unsaturated fatty acids. Under growth-inhibiting conditions, lipid biosynthesis is stopped due to stringent-response regulation, and that is why only growing cells can perform such kind of membrane adaptation [38, 49].

Contrast decrease in membrane saturation can be observed in the presence of polar solvents. Polar solvents are able to incorporate into membrane between the phospholipid headgroups and stimulate the formation of micellar structures. Therefore, microorganisms increase the production of unsaturated fatty acids at the expense of saturated fatty acids. This mechanism was observed in* Escherichia coli* in the presence of ethanol [50].

#### 3.1.2. Isomerization of Unsaturated Fatty Acids

Two different groups of unsaturated fatty acids take part in bacterial adaptation to organic pollutants. Isomerization of* cis* unsaturated fatty acids into correspondent* trans* isomers was described in many papers as adaptation mechanism of the bacterial cells under growth inhibiting conditions [25, 38, 51, 52]. This mechanism is a short-term response triggered in the presence of hydrophobic chemicals that do not need to synthetize new fatty acids.

Various bacterial strains, for example,* Pseudomonas* and* Vibrio,* can adapt to the presence of toxic compounds and their fluidizing properties by isomerization of* cis* unsaturated fatty acids to their appropriate* trans *isomers (Figures 1(b) and 1(c)). These two forms of unsaturated fatty acids have different steric structure. The* cis* configuration of the acyl chain has a nonmovable bend of 30°, which causes steric hindrance and disturbs the highly ordered fatty acid package [51]. In contrast, the steric behavior of* trans* fatty acids and saturated fatty acids is very similar. Nonmovable bends of* trans* fatty acids have 6°. Both* trans* and long-chain saturated fatty acids possess a long extended conformation. It enables them to adopt a denser packing in the cytoplasmic membrane and allows protecting membrane against the fluidizing molecules. That is the reason why the transformation of* cis* to* trans* fatty acid leads to the decrease of membrane fluidity. Another reason for an ordered packing of* trans* fatty acids compared to* cis *isomers is their higher melting temperature (*T*_*M*_).

The studies involving toluene adapted* Pseudomonas putida* strain revealed higher amount of* trans* fatty acids. *T*_*M*_ of cytoplasmic membrane of this strain was 7–9 Kelvin higher in comparison with nonadapted strain. Toluene can decrease lipid ordering with its fluidizing effect and promote the formation of an inverted hexagonal lipid. The observed conversion of* cis *unsaturatedfatty acids into* trans *fattyacids in* P. putida *is expected to counteract the formation of a nonbilayer structure. This observation can be rationalized because the lipid volume of the* trans *fattyacids is smaller than the* cis *isomer [8]. Heipieper et al. [52] proved the activation of the* cis*-*trans* isomerase in resting cells by the addition of 3-nitrotoluene. This activation resulted in the conversion of the* cis* unsaturated fatty acids into the corresponding* trans* isomers. The intensity of the rate of* cis*-*trans* isomerization depended on the added amount of toxic compound. A mutual dependency was found between the activation of this system and the induction/activation of other stress-response mechanisms [53].* Cis*-*trans* isomerization correlates with the toxicity and amount of hydrophobic compound accumulated in cytoplasmic membrane. In most bacteria, the* cis*-*trans* isomerization is conducted by the transformation of oleic and vaccenic acids to their* trans* isomers in consequence of the high prevalence of the acids in cytoplasmic membrane [54].

The enzyme that is responsible for this adaptation mechanism, isomerase, belongs to cytochrome c-type protein and carries Cti polypeptide with a heme-binding site. This polypeptide was found in all tested* Pseudomonas *strains. Moreover, comparison of the amino acid sequences of the seven known Cti proteins identified it as heme containing protein [55]. Cti polypeptide is responsible for the localization of* cis*-*trans* isomerase in periplasmic space. That is the reason why only fatty acids with* cis *double bond in specific depth of membrane can reach the active site of isomerase [56]. This enzyme has been purified from the periplasmic fraction of* Pseudomonas oleovorans* for the first time by Pedrotta and Witholt [57]. The* cis*-*trans* isomerase gene cloned and sequenced from* Pseudomonas putida* P8 [54] and* Pseudomonas putida* DOT-T1E [58] made evident that the isomerase has an N-terminal hydrophobic signal sequence. This sequence is cleaved off after targeting the enzyme to the periplasmic space. The observations confirmed that* cis*-*trans* isomerase is constitutively present, does not require ATP or other cofactors including NAD(P)H and glutathione, and works in the absence of* de novo* synthesis of lipids [20, 49, 59, 60]. The occurrence of heme-binding site of the cytochrome c-type strongly supports a mechanism of* cis*-*trans* isomerization by forming an enzyme-substrate complex. This finding prefers a mechanism for the enzyme, in which electrophilic iron (Fe^3+^), provided by a heme domain, directly attacks* cis* double bond of fatty acid and removes an electron of the* ci*s double bond, thereby transferring the sp^2^ linkage into sp^3^. Double bond is then rebuilt in* trans* configuration.* Cis*-*trans* isomerization is an exergonic (exothermic) reaction because the energetic difference between* cis *and* trans* configuration is 3.1 kJ*·*mol^−1^ [52].

#### 3.1.3. Changes in Cyclopropane and Branched Fatty Acids

Higher concentration of organic pollutants stimulated production of cyclopropane fatty acids (C17-CP and C19-CP) in some bacterial strains. These results were observed under the exposure of polycyclic aromatic hydrocarbons (PAHs), phenols, biphenyl, and polychlorinated biphenyls (PCBs) [7, 28, 61, 62].

The role of these fatty acids in membrane adaptation mechanisms is still not clear because their participation in membrane permeability and fluidity maintenance is not understood in detail [63]. However, it was suggested that the presence of cyclopropane fatty acids in membrane may decrease membrane permeability to protons. Some authors indicated that cyclopropane fatty acid formation is one of the most important mechanisms that protect bacterial cells against many chemicals (aromatic compounds, organic solvents, alcohols, etc.) and environmental factors (salinity, pressure, and temperature) [6, 24, 48, 64–67].

Other authors demonstrated the decrease of the production of these fatty acids after the incorporation of toxic compounds into cultivation media [26, 42, 68]. Perly et al. [69] described poorly packing cyclopropane fatty acids into the acyl chain array of the phospholipid bilayer compared to unsaturated fatty acids. Even low content of cyclopropane fatty acids in cytoplasmic membrane may change overall mobility and order of the acyl chains. These fatty acids are formed from* cis* unsaturated fatty acids under energy consumption. It is thought that the primary function of cyclopropane fatty acids formation is to change chemical properties of the membrane without changes of physical properties [70]. The physiological role of cyclopropane fatty acids in survival of* Escherichia coli* under acid stress conditions was reported [63].

The abundance of branched fatty acids in FAMEs profiles obtained from contaminated environment strains is significantly higher compared to control samples [26, 71].

Lipids of anaerobic and G^+^ aerobic bacteria often contain a high proportion of* iso* and* anteiso* branched fatty acids. Nevertheless, they can be found in several G^−^ strains [26, 28]. The maintenance of membrane fluidity with alteration of branched fatty acids depends on the energetic status of the cells as well as on* de novo *synthesis of their precursors—valine (*iso*-branched-even-chain), leucine (*iso*-branched-odd-chain), and isoleucine (*anteiso*-branched-odd-chain).* Iso* and* anteiso* fatty acids show different physicochemical properties because of the differences in structure and *T*_*M*_ [10, 13, 38, 72, 73]. The *T*_*M*_ of the branched fatty acids is lower for the* anteiso* fatty acids (e.g., 51.7°C for C15:0* iso* and 23.0°C for C15:0* anteiso*) [72]. This difference causes a remarkable change in the fluidity of the membrane when the species of branched fatty acids are changed from one to the other and affect the lipid ordering in particular membrane fraction. The effect on *T*_*M*_ caused by a change from* anteiso*- to* iso*-branching in G^+^ bacteria is comparable to the isomerization of* cis* to* trans* unsaturated fatty acids in G^−^ bacteria. Even the volume occupied with* anteiso* fatty acids is higher than that occupied with* iso* fatty acids. G^+^ and G^−^ bacteria that contain branched fatty acids adapt to differences in temperature and organic solvents by altering the* anteiso/iso* ratio in the cell membrane. According to the different physicochemical properties of those two species of branched fatty acids, the bacteria showed a decreased amount of* anteiso* fatty acids when grown under adverse conditions to decrease the fluidity of membrane and diminish incorporation of the pollutants into membrane structures [28, 62, 73].

Anaerobic sulphate-reducing bacteria contain predominantly* anteiso*-branched fatty acids. The modification of their membrane fluidity is performed by increasing the relative ratio of saturated to* anteiso*-branched fatty acids. Under the growth insufficient conditions, this mechanism does not take place. The growth rate of anaerobic bacteria is much slower compared to that of the aerobic; therefore, the adaptation mechanisms take more time and these bacteria are sensitive to organic compounds to a higher extent than aerobic bacteria [74].

### 3.2. Changes in Phospholipids

**Figure 2:**
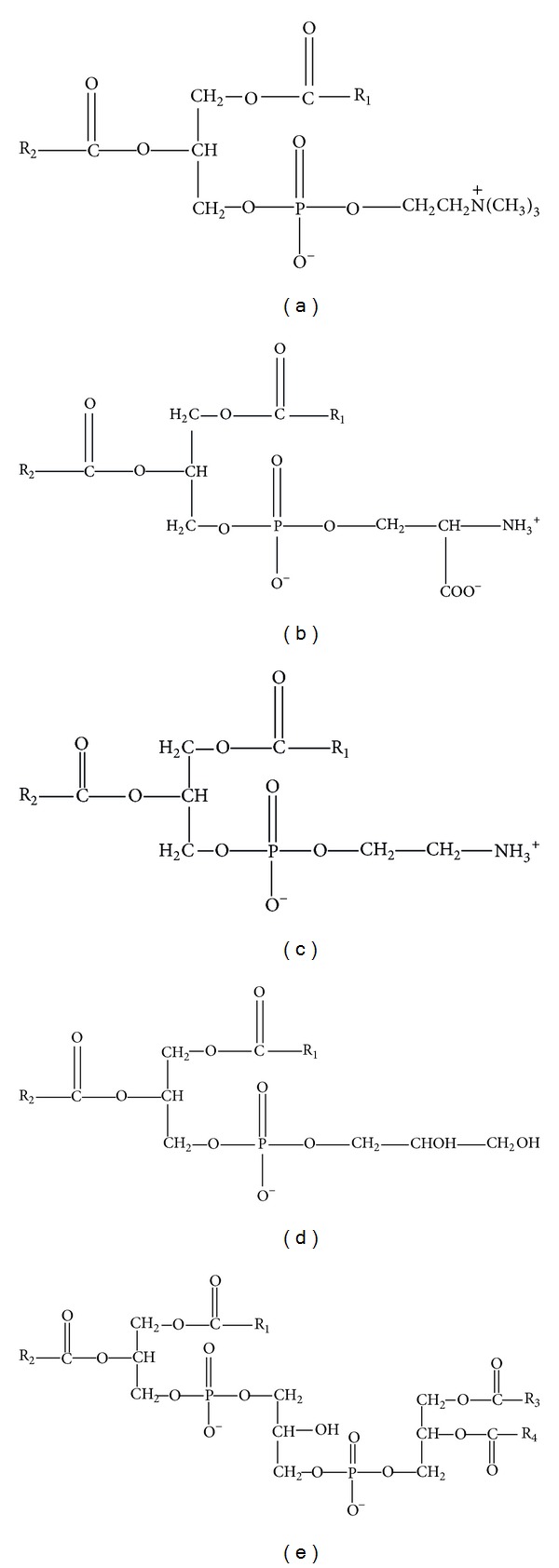
Structure of bacterial membrane phospholipids—phosphatidylcholine (a), phosphatidylserine (b), phosphatidylethanolamine (c), phosphatidylglycerol (d), and cardiolipin (e). R_1_, R_2_, R_3_, and R_4_ represent fatty acid acyl chains.

Bacteria contain in their cytoplasmic membrane several different phospholipid headgroups (phosphatidylserine—PS, PC, PE, PG, and CL) (Figure 2). Each of them holds specific function to maintain vital cell. In the presence of environmental perturbations, cells alter phospholipids amount.

Changes in phospholipids headgroups on environmental pollution are poorly studied compared to fatty acids alteration. Phosphatidylethanolamine (PE) is the most abundant phospholipid in bacterial membrane that comprises more than 70% of all phospholipids [13]. It provides lateral pressure to bacterial membrane bilayer and keeps the position of amino acids. It is a nonbilayer forming lipid because of its steric conformation (small glycerol group and high acyl-chain volume). Nonbilayer aggregates (preferred hexagonal conformation) of cytoplasmic membrane are important in cell division, membrane fusion, and lateral proteins and lipids motion. The ratio between bilayer and nonbilayer forming lipids varies in response to environmental changes. Organic solvents like benzene and toluene can reduce the transition temperature of membrane lamellar-gel to liquid-crystalline phase (*T*_*M*_) and enhance the formation of nonbilayer aggregates with decreasing *T*_*LH*⁡_ temperature (transition from bilayer into hexagonal phase). Stabilization of the *T*_*M*_ temperature is important to sustain membrane fluidity and stability. *T*_*M*_ of cytoplasmic membrane can be slightly modified by membrane phospholipids (each of them has different *T*_*M*_), which can affect bilayer stability of membrane (e.g., dipalmitoyl-PC has 41°C, dipalmitoyl-PE has 63°C, dipalmitoyl-PS has 55°C, and dipalmitoyl-PG has 41°C *T*_*M*_). Cultivation of* Pseudomonas putida* S-12 with toluene decreased amount of PE and increased content of PG and CL. This alteration could stabilize membrane by lowering the fluidity [39]. However, Weber and de Bont [45] described that phospholipids have much higher effect on bilayer stability (*T*_*LH*⁡_) than on membrane fluidity (*T*_*M*_) because of their ability to form hexagonal or lamellar structures. Based on these facts, the decrease of PE content leads to higher bilayer stability. Nevertheless, bacterial cell tries to keep balance between bilayer and nonbilayer phospholipids to maintain its physiological function.

Donato et al. [29] described the effect of DDT on the bacterial strain* Bacillus stearothermophilus*. This compound induced a very significant increase of the PE membrane content with a parallel decrease of PG content. This alteration was accompanied by an increase of straight chains and parallel decrease of branched fatty acids in cytoplasmic membrane. DDT promoted more ordered membrane with an increase of the *T*_*M*_ temperature to higher values that led into higher membrane rigidity. However, increase in PE and decrease of PG amounts are not usual responses of the bacteria. PG is important in CL synthesis and plays a role in protein translocation across the membrane [10].

Based on their polarity, toxic organic solvents can accumulate in different membrane sites. This affects their ability to change membrane bilayer stability by the formation of inverted cone (polar pollutants) or cone structures (nonpolar pollutants). Polar compounds as ethanol can accumulate between the glycerol headgroups. This process destabilizes bilayer-nonbilayer balance. Bacterial cells react to this effect by the formation of a lipid with a small headgroup volume, for example, monoglucosyldiglyceride (MGDG). Benzene, for example, increases hexagonal aggregates. Cells counteract this phenomenon by stimulation of production of lamellar phospholipids (e.g., diglucosylglyceride, DMGM). A similar effect can be observed in the presence of toluene. Toluene is able to incorporate into the membrane between the acyl chains. The cell response is to produce higher amount of CL to stabilize bilayer. CL has a larger headgroup volume compared to PE. The decrease of PE production and increase of CL content will increase the volume of headgroups. This can compensate toluene induced increase of acyl chains volume and stabilize bilayer. Moreover, CL has 10-Kelvin higher *T*_*M*_ than PE. Due to this fact, CL increases membrane rigidity while toluene induces disordering of acyl chains. As mentioned above, an opposite effect occurs in the presence of polar ethanol [68].

The regulation of phospholipid headgroups content controls the ratio between bilayer and nonbilayer membrane structures and the bilayer surface charge density. The accumulation of organic solvents in the lipid bilayer may increase the distance between the lipids in the bilayer. These changes of the phospholipids will affect the surface charge density of the membrane. Similarly, ethanol increases lipids surface area. PS present in cytoplasmic membrane counteracted this effect and provided lower ethanol sensitivity of* E. coli*. PE and PC showed no effect on cell resistance. The explanation for bacterial resistance to environmental stress is an increase in anion-zwitterionic phospholipid ratio observed by Romantsov et al. [75].

**Figure 3:**
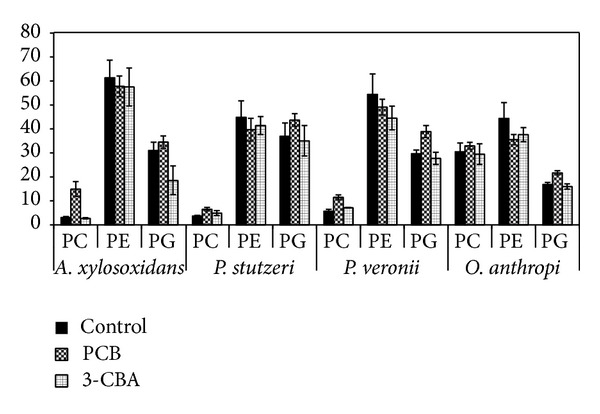
Percentage representation of membrane phospholipids after the addition of nonpolar (PCBs) and polar (3-CBA) toxic pollutants in the presence of four bacterial strains isolated from a long-term PCB-contaminated soil and sediment:* Alcaligenes xylosoxidans, Ochrobactrum anthropi, Pseudomonas stutzeri, *and* Pseudomonas veronii*. PC: phosphatidylcholine, PE: phosphatidylethanolamine, and PG: phosphatidylglycerol.

The effect of nonpolar PCBs and polar 3-chlorobenzoic acid (3-CBA) was assessed in our laboratory using four bacterial isolates. The initial concentration of 100 mg·L^−1^ of each pollutant was added into the minimal mineral media at the beginning of cultivation together with the bacterial inoculum (1 g*·*L^−1^). Adaptation responses in phospholipid headgroups were analyzed after six days of cultivation on the rotary shaker (180 rpm) at 20°C in the dark (Figure 3).

The differences in adaptation responses toward polar and nonpolar toxic compounds can be seen on the examples of PC and PG. Only a minority of bacterial strains contain PC in their membrane [10]. This phospholipid belongs to a bilayer forming group similarly to PG [12]. An increase in PC accumulation in membrane was observed after addition of nonpolar PCBs. Polar 3-CBA did not rapidly affect the amount of this phospholipid in the membrane. Only a slight increase of PC content was observed in both* Pseudomonas* species after 3-CBA addition. On the contrary, both pollutants caused the decrease of PE amount in all studied strains. As mentioned before, PE belongs to nonbilayer phospholipids. Presence of toxic pollutants leads to their accumulation in membrane and destabilizes the bilayer conformation. Cells counteract this effect by reducing the nonbilayer phospholipid fraction to increase membrane stability. This phenomenon was accompanied with an increase in membrane saturation and* cis*/*trans* isomerization to decrease membrane fluidity [28]. Nonpolar compounds are able to accumulate between the acyl chains of phospholipids and stimulate the hexagonal formation and increase *T*_*M*_. As a result of such accumulation, increase of PG content in membrane can be expected. Our results obtained using the PCBs are in accordance with this assumption as can be seen in Figure 3. The presence of 3-CBA caused the decrease of PG content. This can be explained by the ability of a polar compound to accumulate between the polar phospholipid parts (glycerol headgroups) and by a stimulation of micellar formation (interdigitated phase). PG has a larger headgroup volume; therefore, a decrease of the content of this membrane component increases membrane stability. The addition of PCBs evoked increase of PG and PC membrane incorporation and decrease of PE in bacterial cells. Both PC and PG are bilayer forming lipids, while PE is a nonbilayer lipid. These results are in agreement with the results obtained with different nonpolar toxic compounds [45].

#### 3.2.1. Unique Status of Cardiolipin

Cardiolipin (diphosphatidylglycerol) is a unique phospholipid that plays an important role in cell membrane adaptation. Increase in its synthesis strongly enhances the adaptation ability of bacterial cell to the presence of organic solvents as well as to long-term starvation. This mechanism was observed mainly in* Pseudomonas* species [76].

CL is a minor component of bacterial membrane that can be found in many strains. Together with PG, it represents the most abundant anionic lipid component of bacterial membrane. These phospholipids are markedly present in a number of G^+^ bacteria. It may trap protons in an acid structure and bind to a large number of unrelated proteins. The molecule consists of two phosphatidic acid residues linked by a glycerol. It contains four fatty acid chains per molecule and possesses one negative charge per headgroup (Figure 2(e)). CL is synthesized with enzyme cardiolipin synthase in cytoplasmic membrane. The synthase catalyses the transfer of phosphatidyl group between two phosphatidylglycerol molecules and is known as phospholipase D. This enzyme reacts with two PG molecules, one acting as phosphatidyl donor and the other as phosphatidyl acceptor. This enzyme does not have strict substrate specificity and may act in the reverse direction and decompose CL. Trace amount of CL occurs in bacterial cells during the exponential growth phase. Accumulation of CL increases at the beginning of stationary phase. It is the most stable of all membrane phospholipids and is essential for the survival upon long-time starvation. Only* de novo* synthesis of CL was described in bacteria [77]. Prokaryotes can change the amount of this lipid depending on their physiological status and growth conditions.

Increase of the amount of this phospholipid is a known adaptation mechanism in the stress environment. It may reflect a requirement for enhancement of the structural integrity of cytoplasmic membrane or for the support of stress related increases in energy transduction [78]. Another adaptation to long-term exposure to toxic solvent can be achieved with efflux pumps or increased biosynthesis and changes in phospholipids that support the adaptation and repair mechanisms [76]. CL stimulates changes in the physical properties of cytoplasmic membrane. Even small amounts of CL decrease the lateral interaction within the monolayer leaflet, which decreases the energy required to stretch the membrane and could favor the creation of membrane folds [79]. This is the reason why CL is concentrated in polar and septal regions of the cell. It is able to form nonlamellar structures that are required for membrane curvature and lead to the formation of clusters. The advantage of its unique conformation enables the nonlamellar structure to pack tightly forming microdomains which are stabilized by membrane proteins. Its ability to trap protons at H^+^ uptake pathway of energy is due to its high *pK*_*a*_ value (>8) and may have implications for the distribution of the proton-motive force in energy-converting membranes [80, 81].

Recent studies confirmed that bacteria with CL synthase deficiency are more vulnerable to osmotic stress and organic solvents [82]. von Wallbrunn et al. [83] used a mutant bacterium that is not able to synthesize CL to find out whether the* cis-trans* isomerase is able to compensate CL in adaptation mechanisms. Their results demonstrated that the mutant was not able to grow, which proved that* cis-trans* isomerase was not fully able to replace adaptation effect of CL.

### 3.3. Efflux Pumps and Solvent Transport

Bacteria have developed various systems to eliminate toxic compounds naturally present in the environment. This led to the occurrence of multidrug resistance that is a dangerous property of some important pathogens [84]. Such elimination takes place by an uncontrolled efflux and accelerates active extrusion of structurally unrelated compounds from cytoplasm or cytoplasmic membrane to the external space. Toxic pollutants may represent substrates for the efflux system. Several studies indicated the importance of physical properties of compounds (hydrophobicity or molecule charge) for the determination of specificities of this mechanism [85–87]. The efflux system transporters for organic compounds identified in multidrug resistant G^−^ bacteria belong to the RND family (resistance nodulation cell division) of pumps that are encoded chromosomally [88]. This system consists of complex transporters, which export toxic compounds through the cell membranes in a single energy coupled step. It requires a cytoplasmic membrane export system, which acts as an energy-dependent extrusion pump, a membrane fusion protein, and an outer membrane factor [39, 89]. It was found that primary multidrug efflux system AcrAB-TolC facilitated the efflux of hydroxyl-PCBs out of the cells [90]. These multidrug resistant pumps may affect the accumulation and degradation of PCBs by bacteria. Moreover, adapted bacteria of* Pseudomonas* sp. accumulated lower amount of trichlorobenzene in cells than nonadapted strains [76]. Similar results were published with toluene by Segura et al. [91]. The ability of* E. coli* to eliminate PCBs and hydroxyl-PCBs was studied by Geng et al. [90]. The correlation between the multidrug resistance and the efflux of toxic pollutant by* Pseudomonas aeruginosa* was studied in detail by Muller [92]. Rojas et al. [89] described that some of the efflux pumps act on a restricted range of substrates. An example of such pump is TtfDEF pump from* Pseudomonas putida* DOT-T1E, which extrudes only toluene and styrene. Other pumps have a broad range of structurally diverse compounds. MexAB-OprM from* P. aeruginosa *can extrude hexane, p-xylene, and PCBs as well as antibiotics [93].

### 3.4. Proteome

#### 3.4.1. Effect of Pollutants on Membrane Proteins

As a result of the induced change in the membrane lipids, the membrane proteins are affected as well. Adaptation to some organic solvents results in a higher ratio of proteins to phospholipids. This decreases membrane fluidity because of the hindrance of lipid motion with proteins [94]. Cytoplasmic membrane contains mainly solute transport enzymes and proteins involved in electron transport chains. Their activity is changed depending on physicochemical membrane properties (fluidity and bilayer stability) and membrane thickness [9]. Sikkema et al. [21] described the decrease of cytochrome c activity in the presence of polycyclic aromatic hydrocarbons (PAHs). The type of phospholipid headgroup has a pronounced influence on the enzyme activity [95], although the fatty acid composition (lipid ordering) does not affect membrane enzymes. The activity of Ca^2+^-ATPase can be an example. High content of PE increases activity of this enzyme. Some membrane proteins depend on specific boundary phospholipids [45]. Correct orientation and arrangement of specific membrane proteins fully depend on these phospholipids.

#### 3.4.2. Production of Special Proteins

Another known response of bacterial cells is the production and overexpression of stress proteins [96–101]. Induction of stress proteins in* E. coli* with aromatic compounds, such as 2,4-dinitrophenol (DNP) and benzoate, has been reported [102, 103]. Other stress proteins DnaK and GroEL are induced by 2,4-dichlorophenoxyacetic acid in* Burkholderia *sp. YK-2 [104] and by 4-chlorobiphenyl and biphenyl in* B*.* xenovorans *LB400 [97]. Expression regulation of the stress proteins was reviewed by Hecker and Völker [105]. The role of alternative sigma factor *σ* ^B^ in this adaptation was emphasized. This factor controls the production of bmrUR operon in G^+^ *Bacillus subtilis* necessary for the production of multidrug efflux proteins [33]. Toxic environment not only acts on the envelope, but usually affects the cell proteome as well. Damaged proteins can be replaced with the newly synthesized; however, this method is not efficient under nutrient limitations. Therefore, the proteome repair is required to maintain cell vitality. Visick and Clarke [106] described three major mechanisms, which operate in bacteria after a proteome damage induced by environment. First mechanisms include the chaperones, which assist in proper* de novo* folding of proteins and also provide an important means of restoring activity to damaged proteins. Second mechanism describes the existence of enzymatic repair systems that directly reverse certain forms of protein damage, including proline isomerization, methionine oxidation, and the formation of isoaspartyl residues. Third mechanism concerns proteolysis of abnormal proteins, which cannot be repaired.

Martínez et al. [98] described the effect of 4-chlorobenzoic and 2-chlorobenzoic acids on* B. xenovorans *LB400. No effect on membrane lipids was observed. The primary adaptation was revealed as an overexpression of 11 proteins (the highest being the overproduction of catechol-1,2-dioxygenase, belonging to 3-oxoadipate chlorobenzoate degradation pathway). Stress proteins, metabolic proteins, and elongation factors were stimulated as well. Similar induction of metabolic proteins in response to the aromatic compounds was described by Santos et al. [107] and Segura et al. [108]. Ethanol induced production of heat-shock proteins was observed also in* E. coli* [109]. The production of shock proteins belongs to nonspecific general stress responses.

### 3.5. Cell Wall

Cell envelope of all microorganisms consists of cell wall and cytoplasmic membrane. These covering components protect cell nucleus against the outside effects and help in communication with other cells. Most of the adaptation mechanisms discussed in this review are connected with cytoplasmic membrane as highly selective barrier. Moreover, the first line of cell protection is based on the alteration of the membrane composition that leads to lower fluidity and permeability toward toxic compounds. However, cell wall plays also a significant role in cell interior protection. Toxic compounds must firstly penetrate cell wall to reach other cell components. The cell wall of various bacterial strains serves as molecule sieve that prevents the transport of compounds with molecular weight higher than 600–1000 Da [19]. This surface structure is quite dissimilar in G^+^ and G^−^ bacteria. G^+^ bacterial strains have thick murein containing cell wall convoluted with teichoic acids. The role of murein layer in the exclusion of toxic compounds from cell is improbable because of its structure and properties. Mycolata (*Rhodococcus*,* Mycobacterium*,* Nocardia*,* Corynebacterium*,* Gordonia*,* Dietzia*,* Skermania*, and* Tsukamurella*) represent a specific taxon of G^+^ bacteria that are extremely resistant to drugs and toxic hydrophobic compounds. The cell wall of the taxon is unique in its composition and organization compared to other G^+^ bacteria. The dominant abundance has arabinogalactan polysaccharide, which is linked with large 2-alkyl 3-hydroxy branched-chain fatty acids called mycolic acids. This covalently assembled complex is responsible for the cell surface hydrophobicity and impermeability [110–112]. The cell hydrophobic character helps mycolata to uptake the hydrophobic substrate from the environment without the production of surfactant and enables the use of such bacteria in bioremediation technologies [34–36].

Contrarily, G^−^ bacteria have a very thin murein layer that is linked from the outside part with the outer layer. The predominant component of this addition layer is lipopolysaccharide (LPS). LPS is composed of polysaccharide chains with six to seven saturated fatty acids bond in glucosamine disaccharide structure. Thanks to these tightly packed saturated fatty acids, LPS has a very low permeability to hydrophobic compounds and, thus, can act as cell protection [39, 113]. LPS polysaccharide chain plays a role in cell resistance as well. The studies with* E. coli* mutants unable to synthesize these polysaccharides showed high sensitivity of the mutants toward hydrophobic antibiotics, detergents, and other drugs [45].

Moreover, changes in LPS composition led to higher o-xylene resistance of* Pseudomonas putida Idaho*. LPS molecules with high molecular weight were replaced by lower weight bands to adapt to o-xylene [114]. This notion of a protective function of LPS can be supported by a lower sensitivity of G^−^ bacteria toward various organic contaminants such as PCBs, toluene, benzene, or biphenyl [26, 28, 115]. The amount and type of LPS molecules present in bacterial cell wall have a crucial effect on the bacterial surface properties as hydrophobicity and adhesion with outer surfaces and substrates [116]. The decrease of cell hydrophobicity generally leads to lower cell availability toward lipophilic contaminants and diminished permeability [84].

However, some of the adapted microorganisms are able to use the hydrophobic solvents as energy source [117]. Under the circumstances, uptake of such substrates is provided with the release of LPS molecules and enclosing of hydrophobic substrates with hydrocarbon droplets [118]. G^+^ bacterium* Mycobacterium frederiksbergense* showed increase in cell hydrophobicity in the presence of anthracene instead of glucose. Anthracene served as a carbon source for the strain. Bacterium increased the cell surface availability for the metabolic process [31]. Some microorganisms that are capable of utilization of hydrophobic contaminants produce biosurfactants and extracellular mucor to increase bioavailability of such unique carbon sources [119].

Cell survival in inhospitable environment can be supported by the addition of divalent ions (Mg^2+^ and Ca^2+^). It is supposed that these divalent ions can diminish the charge repulsion of adjacent polyanionic LPS molecules with their electrostatic bond. As a consequence, firm ordering of LPS molecules influences membrane stability and the entry of toxic organic compounds. Higher toluene resistance of* Pseudomonas* species was observed after the supplementation of cultivation media with divalent ions [45]. Toluene adaptation correlated with lowered surface hydrophobicity [39]. Higher surface hydrophobicity was observed in the presence of toxic water-soluble arsenical compounds. This effect increased the tolerance of* Euglena mutabilis* and* Euglena gracilis* to hydrophilic toxic compound but not to hydrophobic ones [120]. The removal of LPS molecules can lead to the loss of the resistance to toxic contaminants [113, 121].

Although the penetration of external compounds is diminished by outer membrane, large number of small molecules can move through this cell structure through protein canals. Some of the canals forming proteins are highly specific with specific binding sites facilitating the transport of certain molecules. Other proteins called porins allow nonspecific diffusion, necessary for the nutrient and water influx [87, 115]. Porins are water filled protein canals embedded in the outer membrane. Bacterial resistance toward rather hydrophilic antibiotics is related to the porin mutations [122]. OmpF porin is supposed to be a good transporter for organic solvents [123]. Contrast effect was observed with porin OmpL. Its absence leads to the solvent hypersensitivity, because of its stabilization effect to cell wall integrity [76]. The adaptation to solvents does not only enhance the resistance to other solvents but also enhances the resistance to heavy metals and antibiotics [124].

## 4. Other Adaptation Responses

Another mechanism of microorganism adaptation is the accumulation of special compatible solutes like trehalose, betaine, rhamnolipid biosurfactants, and proline. These solutes were found in cell of stress survived microorganisms because of their ability to protect cell against low temperatures with their effect on lipid ordering and membrane fluidity [125–127]. The solvent accumulation between the phospholipid headgroups increases the membrane fluidity and permeability. As a result, proton leakage can be observed, which leads to the loss of proton-motive force. Bacteria can stimulate the activity of H^+^ ATPase in the presence of high concentration of solvents to decrease the loss of protons [128].

The adaptation of outer membrane is also connected with the formation of outer membrane vesicles [129]. Membrane vesicles play an important role in interspecies communication [130] and the delivery of proteins, toxins, and DNA. Kobayashi et al. [131] discussed the possibility of toluene-containing membrane vesicles as an adaptation mechanism to transport toxic compounds away from the cells. In addition, an important function of membrane vesicles release is their involvement in biofilm formation [132]. Bacteria growing in biofilms are known to be significantly more tolerant to antibiotics, biocides, and other forms of environmental stress [132, 133].

Changes in cell morphology in the presence of toxic compounds were observed in G^−^ [134] as well as in G^+^ bacteria [30]. General responses of G^−^ bacteria to environmental stress were attributed to increase in cell size. G^+^ bacteria showed filamentous growth, increased cell volume, formation of endospores [32], and production of unusual extracellular capsule [135].

Another efficient way of how to cope with toxic compounds is to decrease their toxic effect with their degradation or modification. The degradation enzymes are bond to the inner part of cytoplasmic membrane. The ability of hydrophobic compounds to accumulate in cytoplasmic membrane is minimized with hydroxylation of the compound. The usual degradation pathway begins with the incorporation of hydroxyl group into the pollutant structure [1, 95, 136, 137]. However, increase in pollutant's polarity leads to its higher water solubility and higher availability to the microorganism itself. This situation usually leads to higher toxicity of the environment. Therefore, the microorganisms able to modify toxic compounds try to cooperate with other organisms to achieve complete mineralization of contaminants into CO_2_ and water or at least transform the initial compounds into nontoxic intermediate.

Some molecules present in the nature can help bacteria to survive in the contaminated environment. The mechanisms of these compounds have not been described in detail yet. However, we described that some of these compounds are able to diminish bacterial adaptation mechanisms relating to fatty acids composition [138]. Natural matrices rich in terpene content belong to this group. Many studies described the stimulation effect of ivy leaves, pine needles [139], eucalyptus leaves, tangerine, and orange peel [140–142] to the biodegradation of hydrophobic pollutants. Potential use of synthetic terpenes and natural matrices containing these compounds in the stimulation of bacterial adaptation was studied in our previous works [28, 138, 143]. Our results clearly indicated the positive effect of natural terpene containing matrices, ivy leaves, and pine needles on bacterial survival in the presence of polychlorinated biphenyls (PCBs). Ivy leaves decreased the adaptation responses of* Pseudomonas stutzeri* and* Burkholderia xenovorans* LB400 toward PCBs and increased the biomass growth. Contrast effect was observed in the presence of synthetic terpenes (carvone and limonene) which correspond with other papers [144–148]. Although the positive effects of natural terpenes on bacterial adaptation and survival in the PCB contaminated media have been observed, it is necessary to study the mechanisms of such effect with other toxic contaminants in future works.

## 5. Conclusions and Future Prospects

A number of responses have been observed in bacteria that counteract the effect of organic pollutants. Rigidification of the cell membrane is a consequence of cell adaptation. The alterations in cytoplasmic membrane maintain particular ratio between bilayer and nonbilayer (hexagonal) phospholipids (prevention against the environmentally induced formation of interdigitated structure) and keep the optimal phospholipid ordering to stabilize membrane fluidity. Another mechanism to protect bacterial cell is the efflux of toxic compounds from the membrane bilayer or its degradation. Toxic compounds affect not only cytoplasmic lipids but also cell proteins. This resulted in the development of special protein repair mechanisms by the bacteria. Changes in cell metabolism reflect the degree of pollutant toxicity. Study of these mechanisms is the first step in selection of proper bacterial strains for bioremediation application. Successful environment decontamination requires bacterial strains that are able to degrade particular (one or more) contaminants. Moreover, such strains have to be able to survive and adapt to environmental pollution.

The successful decontamination process use of adapted strains and optimization of bioremediation conditions have been currently extensively studied. The degradation studies in artificial precisely defined matrices (liquid medium, enriched soil, or sediment) and in naturally contaminated matrices under laboratory conditions (microcosms) should be subsequently applied under natural conditions in smaller range (mesocosms).

Another possibility is genetic modification of excellent bacterial degraders to survive under adverse environmental conditions. That allows the degraders to apply their degradation ability (expression of the relevant enzymes) in decontamination process without inhibition of the physiological processes in bacterial cells.
